# Errata

**Published:** 1876-09

**Authors:** 


### ERRATA.

Pape 579: For Dr "E." Curtis Smith read Dr. T. Curtis Smith.

In the same paragraph for "verginam" read "varginam," and for "dittelation," read "dilatation."

Page 580: "Anodine" should be spelled with a "y."

In the prescription, "ergotte" should have the final sylable "æ;" "*feri mar*" should be spelled "*ferri mur.*"

Page 581: In the paragraph on cancer for "the" cancer read "of" cancer.

Page 584: "servix" should be spelled with "c."

Page 585: In fourth line from bottom of page, the letter "e" is omitted in the word "decomposition."

Page 586: In the prescription at top of page for "ozs." in both places read "drs."

In line thirteen below, for "augur" read "sugar."

Page 592: In the fromula of Prof. Hammond, for "grs 1-01" read "1-10."

Page 611: in eleventh line from the bottom of page for "dilte" read "dilute."

Page 628: In the formula for "iodofoami" read "iodoformi."

In the ninth line from bottom of same page for "servation" read "observation."

Page 638: The heads of the two books noticed, viz: "A Manuel of Diet on Health and Diseases," and "On Paralysis from Brain Disease," are displaced; they should appear on page 639, over the respective notices.

Page 640: For "Tressenden" N. Otis read "Fressenden N. Otis."

☞ The Editors are not responsible for the above errors, the printer having failed to submit to them the proof sheets for revision.—[Editors S. M. Record.

